# Closure times of neurocranial sutures and synchondroses in Persian compared to Domestic Shorthair cats

**DOI:** 10.1038/s41598-022-04783-1

**Published:** 2022-01-12

**Authors:** Martin J. Schmidt, Daniela Farke, Carsten Staszyk, Antonia Lang, Kathrin Büttner, Johanna Plendl, Marian Kampschulte

**Affiliations:** 1 Department of Veterinary Clinical Sciences, Small Animal Clinic-Neurosurgery, Neuroradiology and Clinical Neurology, Justus-Liebig-University, Frankfurter Str. 108, 35392 Giessen, Germany; 2 Institute of Veterinary Anatomy, Histology and Embryology, Justus-Liebig-University, Frankfurter Strasse 98, 35392 Giessen, Germany; 3 Unit for Biomathematics and Data Processing, Faculty of Veterinary Medicine, Justus Liebig-University-Giessen, Giessen, Germany; 4 Institute of Veterinary Anatomy, Faculty of Veterinary Medicine, Free University of Berlin, Koserstr. 20, 14195 Berlin, Germany; 5 Department of Diagnostic and Interventional Radiology, University Hospital Gießen, Giessen, Germany

**Keywords:** Preclinical research, Bone imaging

## Abstract

Human-directed selective breeding has modified the phenotype of the modern Persian cat towards an extreme brachycephalic phenotype (‘peke-face’ Persian), which originates from a spontaneous mutation that first appeared in the 1950s in traditional Persian types. It was suggested that the peke-face phenotype results from pathologic skull development and might represent a craniosynostosis of the coronal sutures. We followed this hypothesis and investigated the time dependent status of the neurocranial sutures and synchondroses in an ontogenetic series of doll-faced and peke-faced Persian cats compared to Domestic Shorthair cats (DSHs). Cranial suture closure was assessed by examining an ontogenetic series of formalin-fixed head specimens (n = 55) and dry skulls (n = 32) using micro-computed tomography. Sagittal, metopic, coronal and lambdoid sutures as well as intersphenoidal, spheno-occipital and spheno-ethmoid synchondroses were examined. Logistic regression analysis was performed to test the global effect of age on suture closure within a group of peke-face Persians, doll-face Persians and DSHs and the 50% probability of having a closed suture was calculated and compared between groups. Age was a perfect predictor for the condition of the coronal sutures in peke-face Persians. Coronal sutures were found to be closed at 0–0.3 months. In doll-face and DSHs, coronal sutures were open throughout the lifetime with the exception of a few very old cats. Results of this study confirmed a coronal craniosynostosis that likely causes the extreme brachycephalic skull morphology in the peke-face Persian.

## Introduction

Human-directed selective breeding has modified the modern Persian cat towards an extreme brachycephalic phenotype with a fundamentally altered head morphology including an extremely round, dome-shaped head, protruding eyes and greatly reduced facial bones resulting in the forehead, nose and jaws being in vertical alignment when viewed from the side^1,2^. This type was called ‘peke-face’ Persian by breeders, which refers to the highly brachycephalic Pekingese dog^1^. The modern phenotype was reported as originating from a morphological variation of traditional Persian types (so called ‘doll-face Persian’) that first appeared in the 1950s^1,2^. The ‘peke-face Persian’ widely displaced the traditional ‘doll-face Persian and dominates Persian breeding lines worldwide, although highly brachycephalic phenotypes are associated with severe health problems in cats, with a negative impact on their quality of life^3,4^. The shortened snout constricts the nasal passages^5^ and can result in respiratory distress^4,6,7^. Due to the reduction of the maxillary alveolar space, the teeth are positioned at abnormal angles and overlap each other, causing dental and gingival problems^2,6,8,9^. Severely bulging eyes (exophthalmos) is a consequence of shallow orbits^5^ and increases the predisposition for corneal ulcerations^8,9^. It was also shown that with the greater reduction of the facial bones, deformation of the neurocranium also increases^10^. With increased reduction of the snout, the neurocranium also gets shorter but wider which results in a significantly reduced cranial capacity in peke-face Persians leading to cerebellar herniation, intraforaminal crowding and associated obstructive internal hydrocephalus^10^. The question arises, as to whether the profound phenotypic modification of the skull is part of a physiologic spectrum of skull phenotypes in the Persian breed or rather reflects pathologic skull development. A previous investigation of the skull of Domestic Shorthair (DSH) and Persian cats noted absent coronal sutures in young peke-face Persian kittens^10^. It was hypothesised that the skull conformation of this Persian type may result from premature closure of this suture, but this theory was based on the observation of only five animals and might have been incidental. Knowledge concerning physiological suture closure in cats is also quite limited. We therefore examined the time dependent status of the neurocranial sutures and synchondroses in an ontogenetic series of doll-faced and peke-faced Persian cats compared to DSH following the hypothesis that peke-face Persians undergo premature closure of the coronal sutures.

## Materials and methods

### Data acquisition

Cranial suture closure was assessed by examining an ontogenetic series of formalin- fixed head specimens (n = 55) and dry skulls (n = 32). The heads and skulls of 87 cats of different ages (45 Domestic Shorthair and 42 Persian cats), were collected from the Department of Veterinary Clinical Sciences, Clinic for Small Animals and from the Clinic for Gynaecology, Andrology and Obstetrics of the Justus-Liebig-University, Giessen (Table 1). The cats were euthanised or died due to diseases unrelated to the skull and central nervous system. Euthanasia was performed by intravenous injection of 150 mg/kg pentobarbital (Narcoren^®^). Informed consent was obtained from all owners who donated their animals for the study and actual cats remained anonymous. In addition, Persian breeders cooperated with the study and sent in kittens that died in the first weeks of life. Furthermore, Veterinary Anatomical Institutes of the University of Berlin and Leipzig made dry cat skulls from their collections available for the study (all immature DSHs). Head and skull specimens of 45 DSHs were examined (group 1). Their ages ranged between 1 day and 240 months (mean 64 months; SD 77.6). All specimen in the group of Persian cats were formalin-fixed whole skull preparations. From 42 Persian cats, 12 had a doll-face morphology (group 2). Their ages ranged between five months and 235 months. Their mean age (75.3 months; SD 76.8) was higher than the mean age of peke-face Persians (p = 0.015) but not compared to DSHs (p = 0.67). Four of the doll-face Persians were female and eight were male. Thirty Persians had a peke-face morphology (group 3). The age of cats in this group ranged between one day and 181 months (mean 25.5 months; SD 47.4). Peke-face Persians had a significantly lower mean age (p = 0.0177) than DSHs. Fourteen cats were male, 16 were female.

**Table 1:** Overview of the open or closed status in the examined MCTs in the different groups of cats.

Suture	Domestic Shorthair cat (n = 45)		Peke-face Persian (n = 30)		Doll-face Persian (n = 12)	
Status	Open	Closed	Open	Closed	Open	Closed
Coronal suture left	42	3	0	30	9	3
Coronal suture right	41	4	0	30	9	3
Sagittal suture	26	19	8	22	12	0
Interfrontal (metopic) suture	39	6	21	9	9	3
Lambdoid suture right	40	5	26	4	9	3
Lambdoid suture left	39	6	26	4	9	3
Spheno-occipital synchondrosis	24	21	26	4	4	6
Intersphenoidal synchondrosis	25	20	16	14	4	6
Spheno-ethmoid synchondrosis	24	21	15	15	0	12

### Skull analysis and determination of general head morphology in Persian cats

Persian cats were grouped based on their skull features as described previously^10^. Three-dimensional head models were created based on CT data sets with a threshold tool that enabled visualisation and selection of the skin surface. A transversal ortho‐slice plane was aligned on the rostral end of the eye‐globes in the 3D model. If the tip of the nose was on or caudal to that plane, the cat was assigned to the peke‐face group. If the tip of the nose was rostral to that line, the cat was classified as a doll‐face Persian.

### Micro‐CT (MCT)

Dry skulls and whole head specimens were examined using a MCT System Skyscan 1173 (Bruker MicroCT, Kontich, Belgium). Heads were scanned with a tube voltage of 130 kVp and a tube current of 60 μA. The image pixel size was set to 14.74 μm (nominal isotropic resolution). All samples were scanned over 180° (plus fan angle) in rotation steps of 0.2° and a four‐fold frame averaging for noise reduction. To reduce image artefacts, beam hardening was carried out with a brass filter of 0.25 mm thickness. Cross‐sectional images were reconstructed based on a cone‐beam reconstruction algorithm using the NRecon Reconstruction Software (version 1.6.10.2). A Gaussian kernel (σ = 2) was taken for image noise reduction. The 3D volume rendering was performed using the ANALYZE software package.

### Image analysis

**Figure 1:**
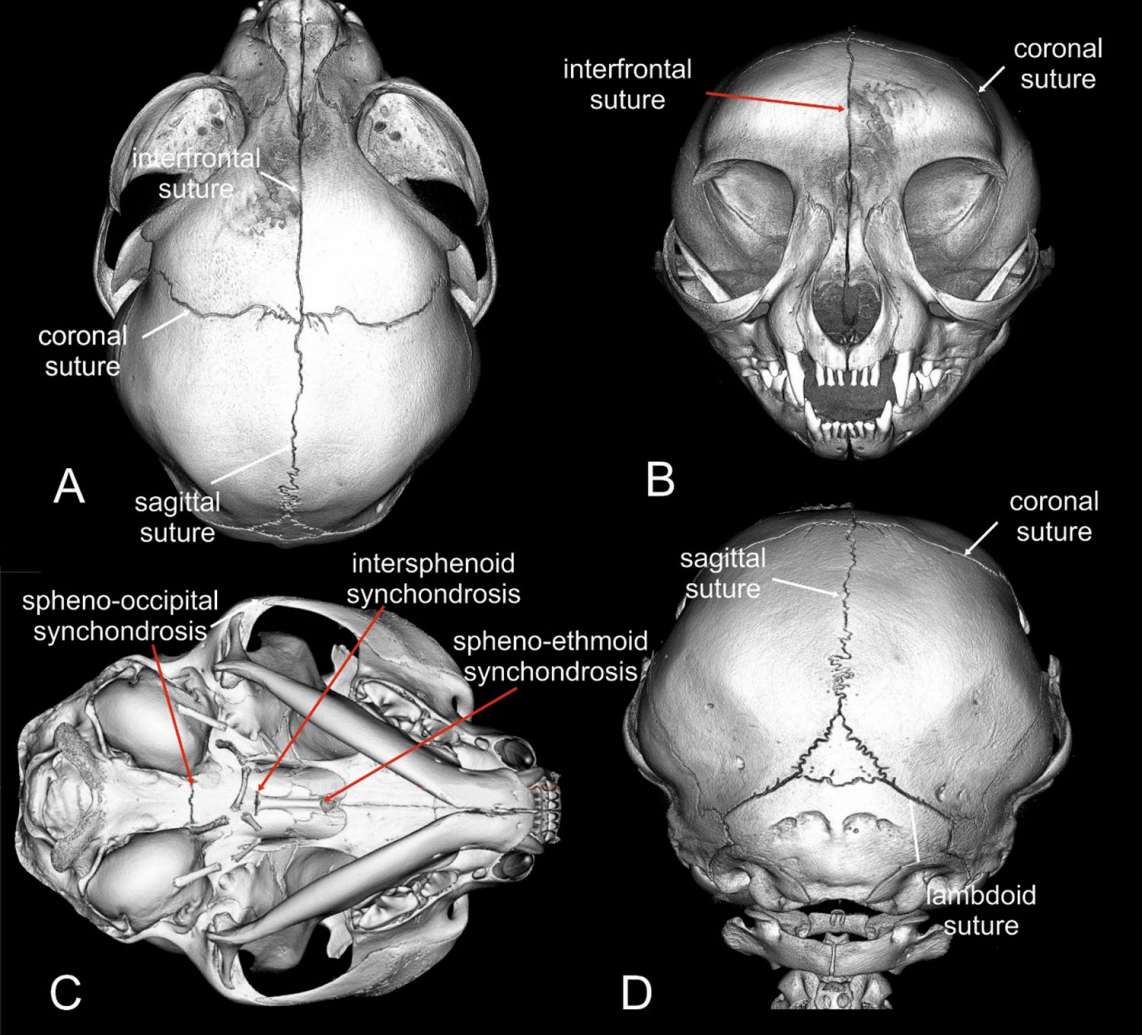
3D head models based on micro-CT images of a 6-month-old Domestic Shorthair cat in a dorsal (**A**), frontal (**B**), ventral (**C**) and caudal view (**D**) displaying the examined sutures and synchondroses.

**Figure 2:**
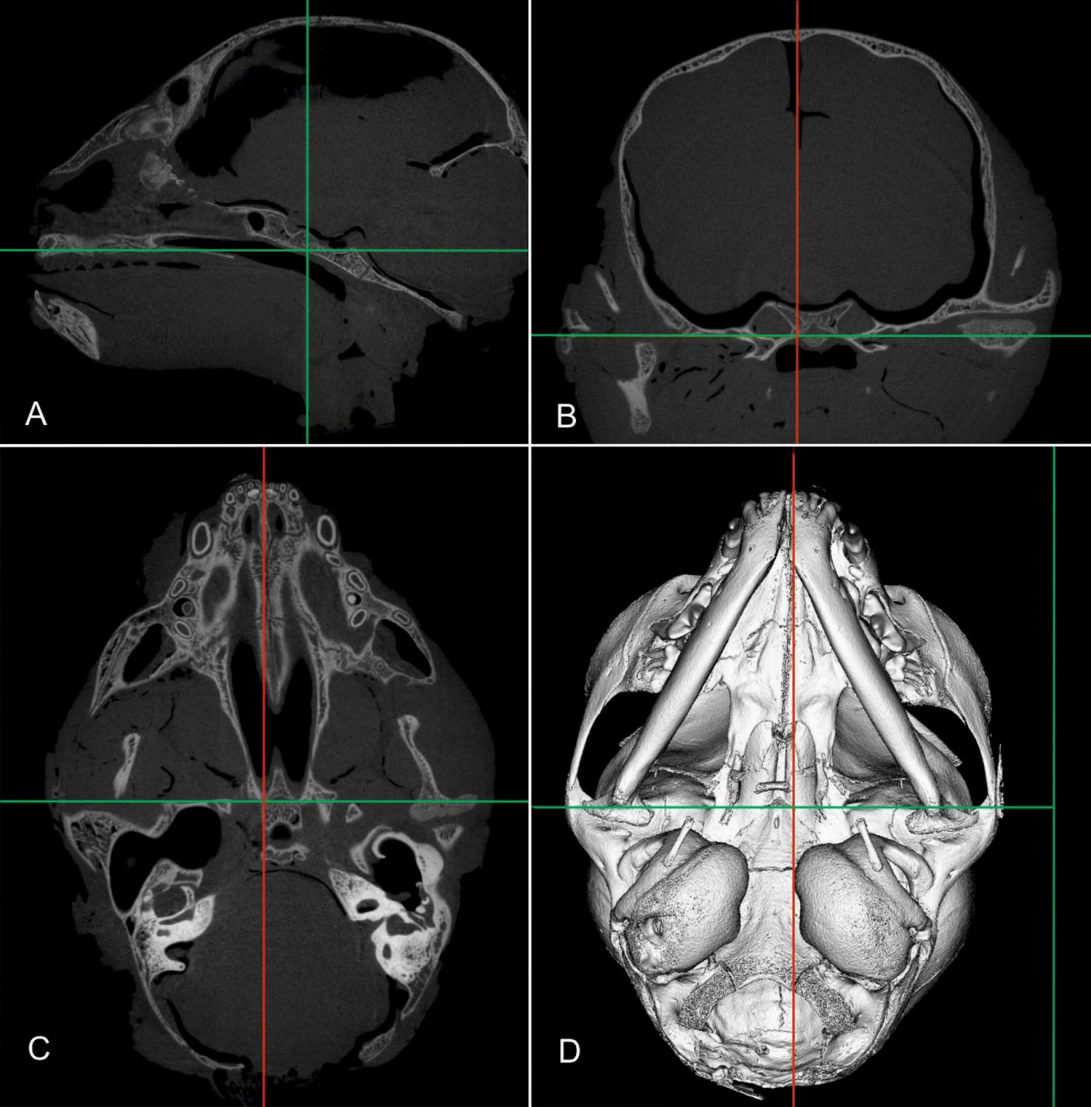
Combination of micro-CT images and a 3D head model of a 6-month-old Domestic Shorthair cat in a sagittal (**A**), transverse (**B**) and dorsal plane (**C**) in ANALYZE.

**Figure 3:**
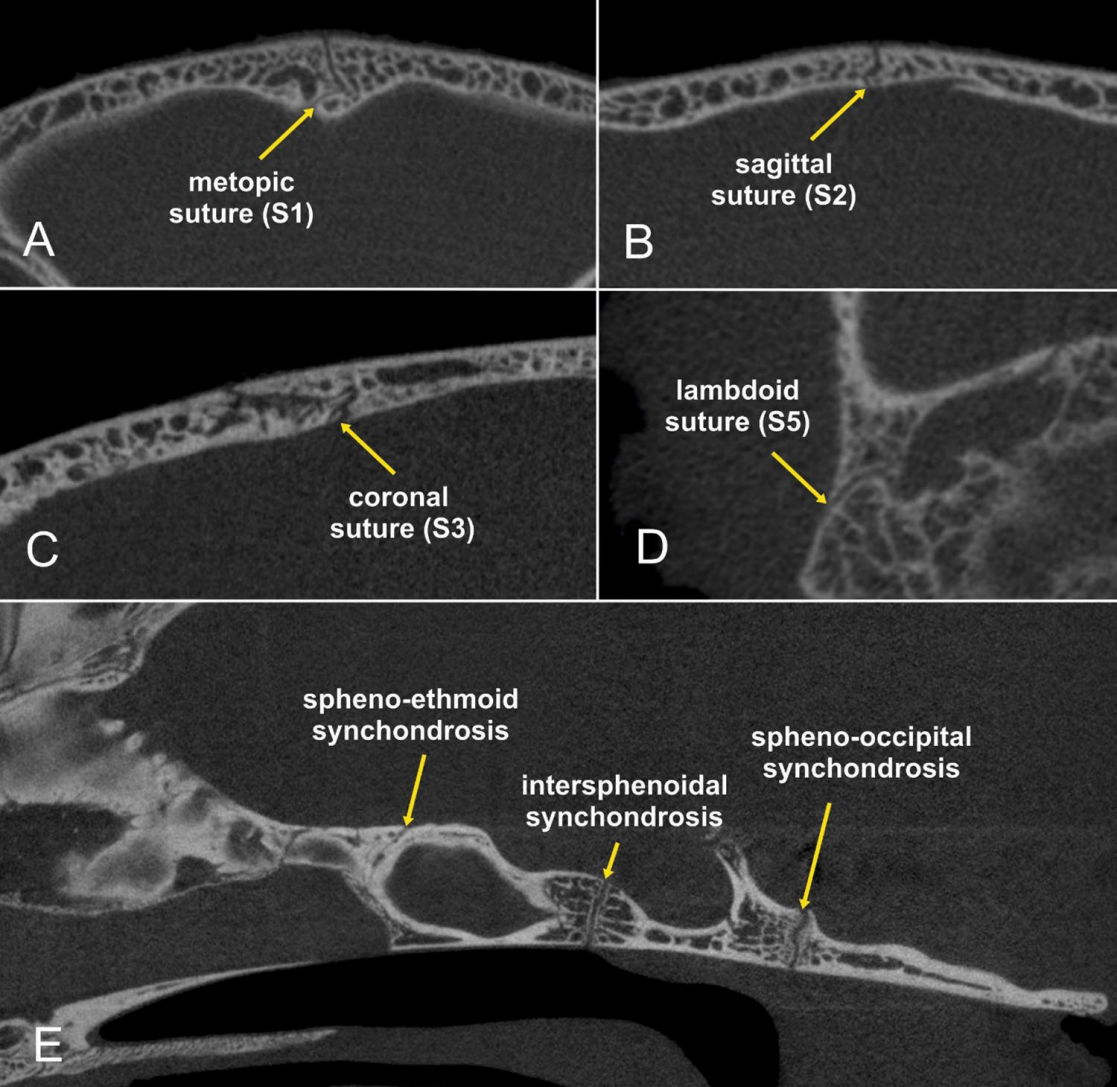
Micro-CT images of a skull specimen of a 6-month-old Domestic Shorthair cat in a transverse (**A**,**B**), sagittal (**C**,**E**) and dorsal view (**D**). The presented sutures and synchondroses separate the adjacent skull bone by a hypodense line of variable shape. The two ends of the coronal suture overlapped (sutura squamosa) leaving thin sutural gaps. Straight-edged plane suture ends were found in the lambdoid suture. The sagittal and metopic sutures had a serrated morphology, with the bone edges having a saw-like appearance. Synchondroses were visible as large, straight-edged or curved hypodense gaps between the basioccipital and sphenoid bone (spheno-occipital synchondrosis), the basisphenoid and presphenoid bone (intersphenoid synchondrosis) and the presphenoid and ethmoidal bone (spheno-ethmoidal synchondrosis).

Investigations were performed using anonymised and randomised image data sets. The observers were blinded to age and breed of cat. Patency or fusion of cranial sutures and synchondroses was assessed using the ANALYZE software package. Window levels, window widths and magnification were adjusted as needed in order to optimise visualisation of bone. The following cranial growth centres were examined: sagittal (S1), interfrontal (metopic; S2), left and right fronto-parietal (coronal; S3/S4) sutures, and left and right lambdoid sutures (S5/6) (Fig. 1), as well as intersphenoidal synchondrosis (S7), spheno-occipital synchondrosis (S8) and spheno-ethmoid synchondrosis (S9) (Fig. 1). Each suture and synchondrosis was examined in both 2D MCT slices in orthogonal views and on the surface of the 3D skull models. Using the graphical software, landmark lines were set on the area of interest in the skull model. The same landmarks were shown in all 2D planes supporting the correct identification of a suture or synchondrosis (Fig. 2). In each specimen, the state of the sutures was coded as either open or closed. Sutures were viewed across the entire length of the suture in question. Sutures and synchondroses that were partially fused were classified as closed. An open synchondrosis or suture was defined as a hypodense, non-calcified zone between well-defined hyperdense borders of the calvaria or cranial base bones (Fig. 2). A closed suture or synchondrosis was defined as a continuous hyperdense bone signal, without interruption by a hypodense zone (Fig. 3).

The 3D model is seen from a ventral view (D). Landmark lines can be set on the area of interest in the model. The same landmarks are shown in all 2D planes supporting the correct identification of a suture or synchondrosis.

### Histological sample preparation

In addition to the CT analysis, the sutures and synchondroses on the heads of 12 cats (2 immature/2 adult DSHs; 2 immature/2 adult peke-face Persians; 2 immature/2 adult doll-face Persians) were histologically examined. After post-mortem scanning, soft tissues were carefully removed from the skull and the whole head was immersion fixed in 10% neutral buffered formalin. Sutures and synchondroses were cut out from each head in 1–2 cm^2^ blocks using a diamond coated sawblade. Suture morphology was assessed in a plane of sectioning perpendicular to the individual suture orientation. Samples were washed with 1% phosphate buffered saline and were decalcified over four weeks in 10% ethylenediaminetetraacetic acid (EDTA) at pH = 8. After decalcification, specimens were embedded in paraffin wax, serial sections of 5 μm were cut and then stained with haematoxylin and eosin and Masson–Goldner trichrome in order to visualise bone, cartilage and fibrous connective tissue. Histological sections were viewed under a Leica DMLB photomicroscope (Leica Microsystems: Wetzlar, Germany).

### Statistical analysis

All statistical tests were selected and performed by a professional statistician (KB), using the commercially available statistic software package SAS^®^. As animal age was not normally distributed, it was log10-transformed.

In a first step, a logistic regression analysis was performed to test the global effect of age on suture closure within the groups of peke-face Persians, doll-face Persians and DSHs by means of a Wald test and using a chi-square statistic. For some sutures, a complete separation for the categories ‘open’ or ‘closed’ according to the perfect predictor ‘age’ was found, which is why a logistic regression model could not be fitted in these cases. For sutures, in which a dependency of age in the logistic regression model was found, a logistic probability model was used to predict the age at which 50% of the cats had a closed suture within each group. Furthermore, logistic regression was used to determine odds ratios (ORs) for having a closed suture with each month of life. In a second step, logistic analysis was performed for sutures S1–S6 in order to determine the effect of age on suture closure as well as testing the impact of the groups of DSHs and doll-face Persians. Only sutures/synchondroses S1–S6 were analysed as synchondroses S7–S9 showed complete separation according to age in at least one of the groups. For all tests, a P value of < 0.05 was determined to be significant.

### Ethics approval and consent to participate

Specimens were examined with approval of the owners that donated dead animals for the study. Studies on dead animals are not subject to institutional or governmental regulations in Germany.

### Consent for publication

All authors approved publication of the manuscript.

## Results

**Figure 4:**
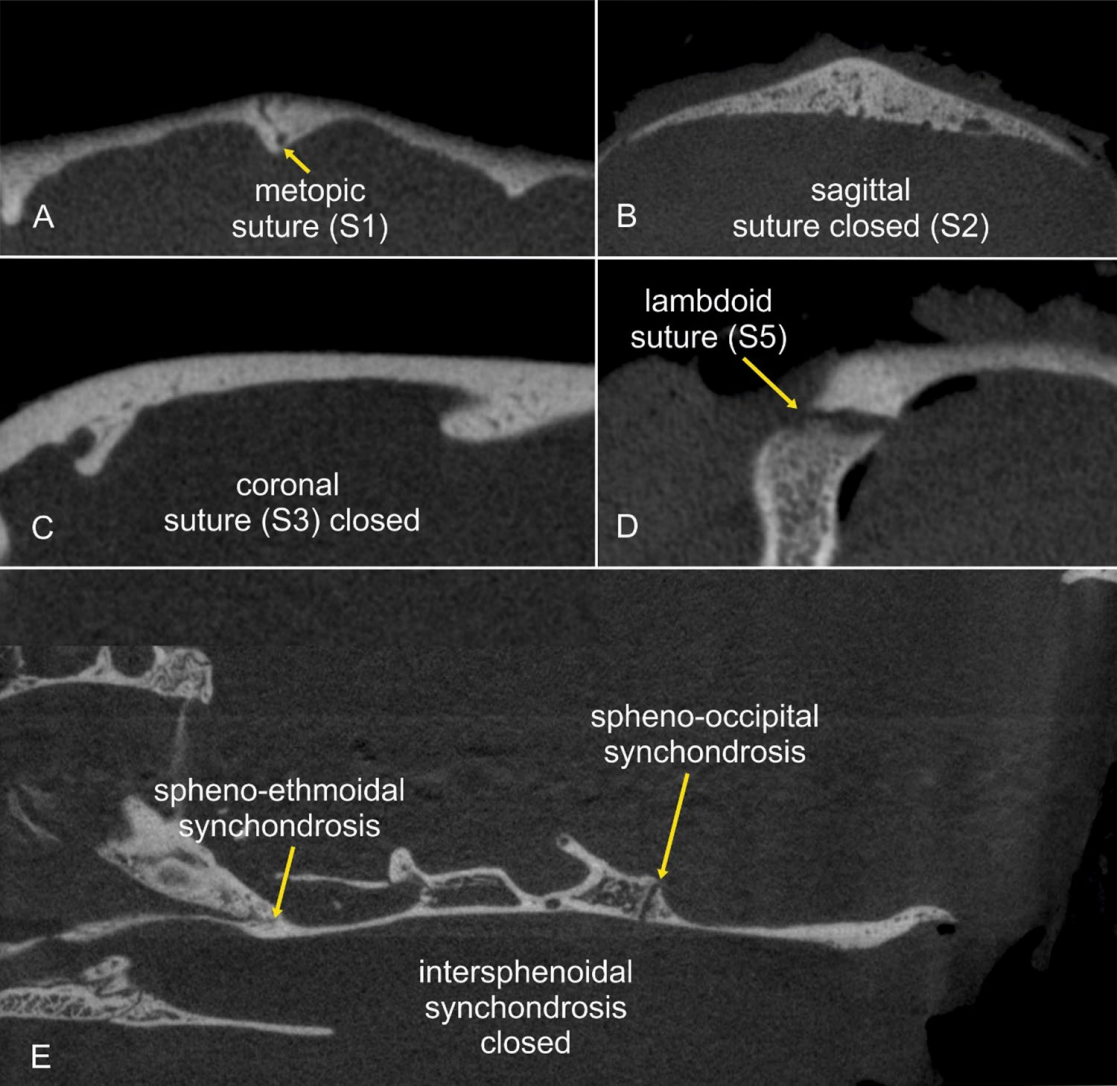
Micro-CT images of a skull specimen of a 12-week-old peke-face Persian cat in a transverse (**A**,**B**), sagittal (**C**,**E**) and dorsal view (**D**). The synchondroses separate the adjacent skull bone by a hypodense line of variable shape. The coronal and sagittal sutures are fused.

**Figure 5:**
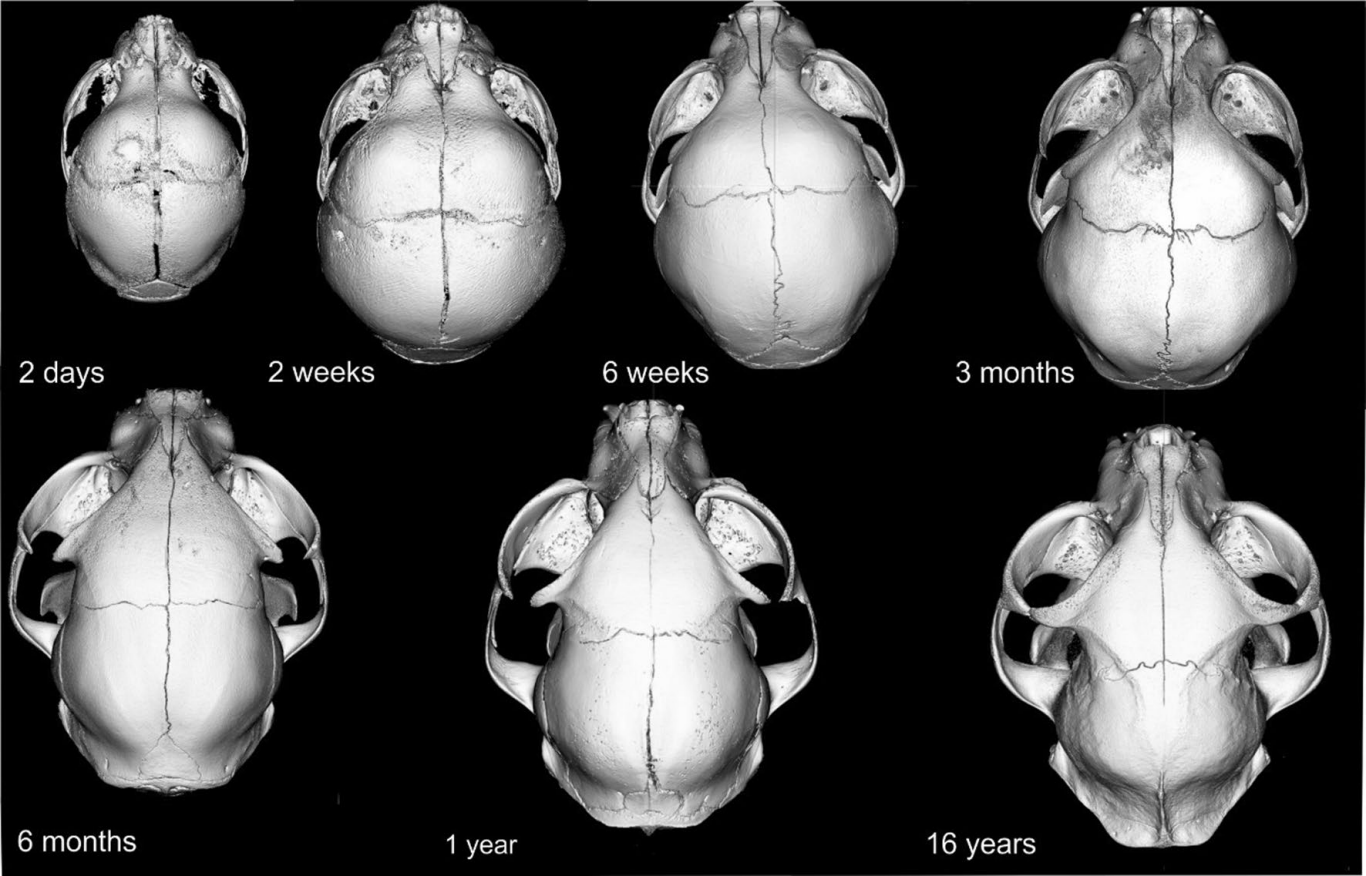
3D models of a series of skull specimens based on micro-CT images of Domestic Shorthair cats at various ages in a dorsal view.

**Figure 6:**
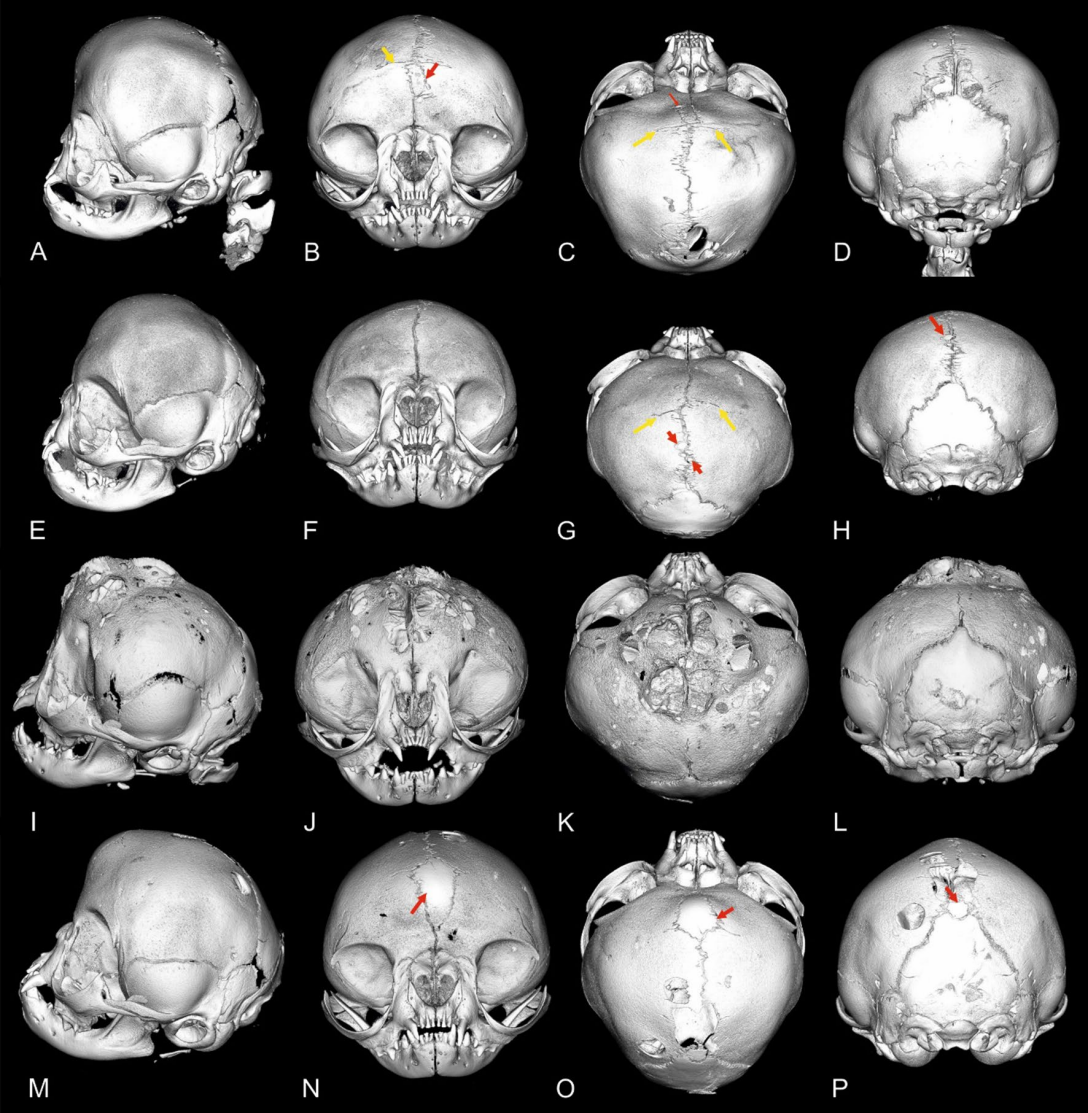
3D models of a series of skull specimens based on micro-CT images of four peke-face kittens. Red arrows highlight intrasutural ossification centres (Wormian bones). Yellow arrows indicate the remnants of coronal sutures, which are mostly closed.

Micro-CT analysis allowed the assessment of suture status and morphology in all examined heads. Individual suture morphology could be clearly visualised (Figs. 3, 4). The open and closed status of sutures and synchondroses are summarised in Table 1. While the calvarial sutures were mostly open in the DSHs and doll-face Persians, a high number of ossified coronal and sagittal sutures was found in peke-face Persians (Fig. 5). Bone deposition occurred along the fused sagittal sutures producing an internal and external bulge. Non-ossified calvarial sutures appeared diastatic with a number of secondary intrasutural ossification centres (‘Wormian bones’) in 12 peke-face Persians. Large irregular defects were seen in the frontal and parietal bones. The interparietal bone appeared subjectively large in relation to the skull compared to the DSHs. In two peke-face kittens, cranial contents bulged dorsally, giving rise to a trilobate shape (Fig. 6).

In the DSH group, results of logistic regression analysis revealed a significant influence of the variable ‘age’ on the open or closed status of the sagittal suture, as expected (S1) (p = 0.0003). The effect of the variable ‘age’ was positively directed (i.e. with increasing age the probability of closure also increased). Analysis of the OR revealed a 16 times increased chance for a closed sagittal suture (p = 0.0003) for each month of postnatal ontogenesis. The other examined calvarial sutures were all unfused, which is why an influence of age was not found. The effect of age was also significant for the condition of the spheno-ethmoid (p = 0.0004) and intersphenoid synchondroses (p = 0.0006) (Table 2). The OR revealed a 38 (S7) or 59 times (S9) increased chance of having a closed synchondrosis for each month of postnatal life. The age at which the suture/synchondrosis had a 50% probability of being closed in the different groups is summarised in Table 3. Age was a perfect predictor for the condition of the spheno-occipital synchondrosis, which closes between 18 and 29 months in DSHs.

**Table 2:** Overview of the results of logistic regression analysis to determine the 50% probability of having a closed suture/synchondrosis. *CS* complete separation, *DSH* Domestic Shorthair cat, *S1* sagittal suture, *S2* metopic suture, *S3* left coronal suture, *S4* right coronal suture, *S5* left lambdoid suture, *S6* right lambdoid suture, *S7* intersphenoid synchondrosis, *S8* spheno-occipital synchondrosis, *S9* spheno-ethmoid synchondrosis.

Group	Suture	Intercept	Standard error	Regression coefficient	Standard error	Log age 50%	Age (months)
DSH	S1	− 4.08	1.31	2.81	0.78	1.45	28.46
DSH	S2	− 3.71	1.35	1.32	0.69	2.80	644.06
DSH	S3	− 3.90	1.5	1.19	0.77	3.27	1884.15
DSH	S4	− 3.95	1.55	1.07	0.80	3.67	4778.42
DSH	S5	− 5.07	2.16	1.92	1.06	2.64	438.48
DSH	S6	− 3.40	1.20	1.14	0.63	2.96	913.06
DSH	S7	− 5.11	1.68	3.63	1.03	1.40	25.41
DSH	S8	CS	CS	CS	CS	CS	CS
DSH	S9	− 5.43	1.81	4.09	1.19	1.32	21.28
Doll-face	S1	− 0.71	0.43	− 0.07	0.38	− 9.79	0
Doll-face	S2	− 0.96	0.45	− 0.42	0.43	− 2.27	0.01
Doll-face	S3	CS	CS	CS	CS	CS	CS
Doll-face	S4	CS	CS	CS	CS	CS	CS
Doll-face	S5	CS	CS	CS	CS	CS	CS
Doll-face	S6	CS	CS	CS	CS	CS	CS
Doll-face	S7	− 0.48	0.43	0.43	0.38	1.11	13.18
Doll-face	S8	CS	CS	CS	CS	CS	CS
Doll-face	S9	CS	CS	CS	CS	CS	CS
Peke-face	S1	− 7.39	4.68	3.85	2.34	1.91	82.66
Peke-face	S2	− 7.89	5.50	3.82	2.68	2.06	115.92
Peke-face	S3	− 6.96	4.67	3.36	2.3	2.07	117.64
Peke-face	S4	− 7.9638	6.06	3.58	2.9	2.22	167.19
Peke-face	S5	− 2.9585	2.21	1.26	1.19	2.33	215.09
Peke-face	S6	− 2.6073	2.10	1.07	1.14	2.43	273.32
Peke-face	S7	CS	CS	CS	CS	CS	CS
Peke-face	S8	CS	CS	CS	CS	CS	CS
Peke-face	S9	CS	CS	CS	CS	CS	CS

**Table 3:** Overview of minimum and maximum times in which the sutures and synchondroses were open or closed. *S1* sagittal suture, *S2* metopic suture, *S3* left coronal suture, *S4* right coronal suture, *S5* left lambdoid suture, *S6* right lambdoid suture, *S7* intersphenoid synchondrosis, *S8* spheno-occipital synchondrosis, *S9* spheno-ethmoid synchondrosis.

Suture	Group	Minimum age open (months)	Maximum age open (months)	Minimum age closed (months)	Maximum age closed (months)	Age (months) of 50% probability to have a closed suture/synchondrosis
S1	Doll-face Persian	5	235	88	186	
S1	DSH	0.03	216	5	240	
S1	Peke-face Persian	0.03	86	0.03	181	
S2	Doll-face Persian	5	235	88	186	
S2	DSH	0.03	240	5	216	
S2	Peke-face Persian	0.03	181	0.03	7	
S3	Doll-face Persian r	5	235	88	186	
S3	DSH	0.03	240	5	210	
S3	Peke-face Persian			0.03	181	Complete separation
S4	Doll-face Persian	5	235	88	186	
S4	DSH	0.03	240	5	210	
S4	Peke-face Persian			0.03	181	Complete separation
S5	Doll-face Persian	5	235	14	186	
S5	DSH	0.03	240	29	210	
S5	Peke-face Persian	0.03	86	108	181	Complete separation
S6	Doll-face Persian	5	235	10	186	
S6	DSH	0.03	240	5	210	
S6	Peke-face Persian	0.03	86	108	181	Complete separation
S7	Doll-face Persian	5	10	14	235	Complete separation
S7	DSH	0.03	138	8	240	
S7	Peke-face Persian	0.03	86	0.75	181	
S8	Doll-face Persian	5	10	14	235	Complete separation
S8	DSH	0.03	18	29	240	Complete separation
S8	Peke-face Persian	0.03	86	108	181	Complete separation
S9	Doll-face Persian			88	235	Complete separation
S9	DSH	0.03	102	5	240	
S9	Peke-face Persian	0.03	3	4.5	7	Complete separation

**Figure 7:**
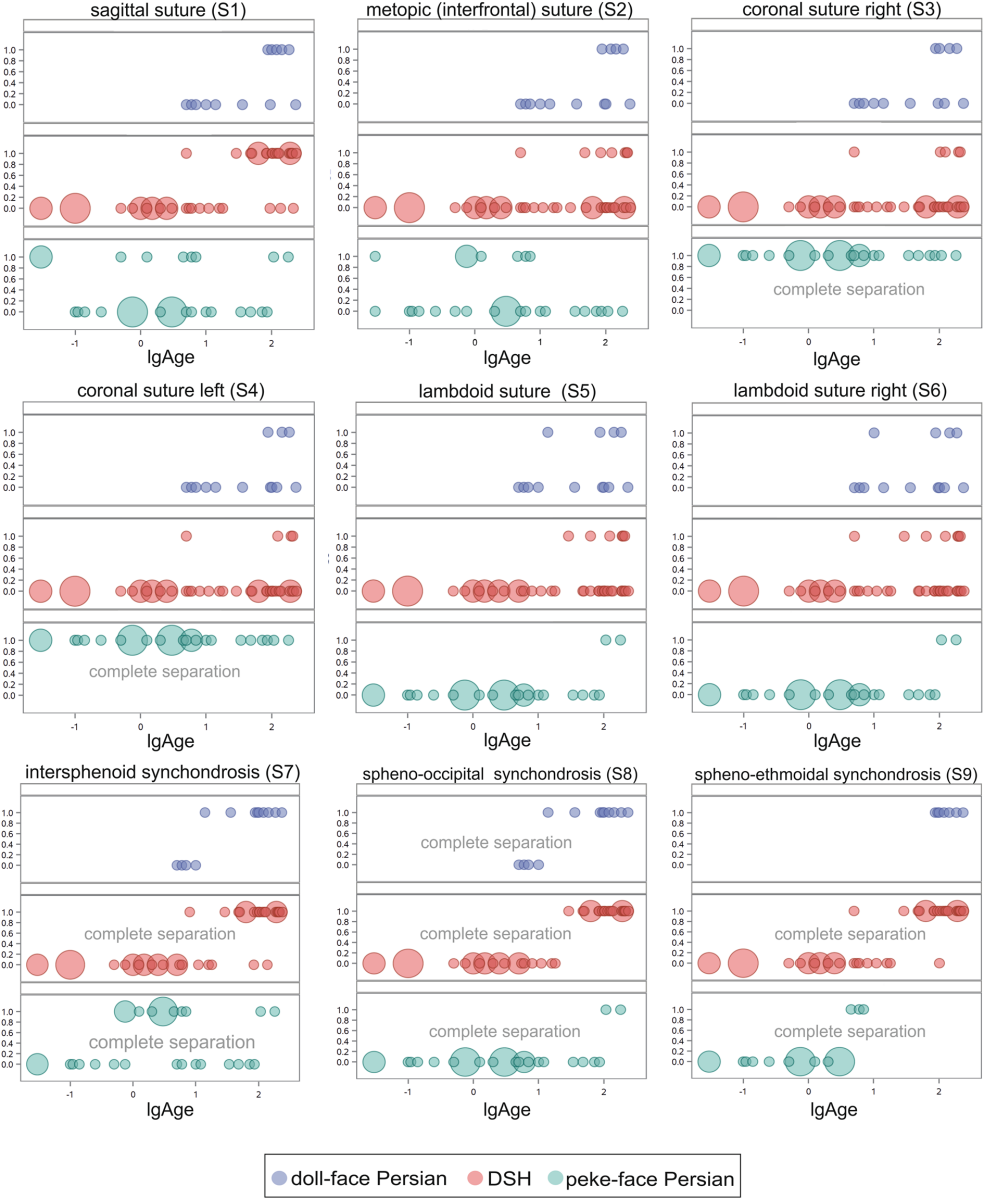
Scatter plot of the open and closed status of the examined sutures and synchondroses in the different groups depending on age. Domestic Shorthair cats indicated in red circles, doll-face Persians in blue and peke-face Persians in green circles. Circle sizes indicate number of animals.

In the group of peke-face Persians, the statistical analysis revealed a complete separation according to age for both coronal sutures (S3 and S4) (age 0–0.03 months = neonate) and the lambdoid sutures (S5/S6) that close between 86 and 108 months (Fig. 7). The influence of age on closure of the metopic and sagittal sutures was not significant. Age was a perfect predictor for the condition of the intersphenoidal synchondrosis (S7) that closes between 86 and 108 months, and the spheno-ethmoid (S8) and spheno-occipital synchondroses (S7) that both close between 10 and 14 months.

In the group of doll-face Persians, results of logistic regression analysis revealed no significant influence of the variable ‘age’ on the open or closed status of the calvarial sutures. Calvarial sutures were mostly unfused until old age. Age was a perfect predictor for the condition of the intersphenoidal, (S7), spheno-ethmoid (S8), spheno-occipital synchondroses (S9) that close between 10 and 14 months. Regression coefficients (i.e. slope of the regression curves) were unequal between groups, which is why determination of significant differences between the time points of different groups for the 50% probability of having a closed suture/synchondrosis was not possible.

### Histomorphology of the sutures and synchondroses

In unfused calvarial sutures, the gap between the bony edges was filled with collagen fibre bundles interspersed with elongated fibrocytes (Supplementary Fig. 1). In young cats, the sutures contained loosely arranged connective tissue showing a collagen fibre orientation preferentially parallel to the sutural alignment (Supplementary Fig. 2). The sutures were continuous with the periosteum and endosteum. These sutures also featured high amounts of osteoblasts and fibroblasts (Supplementary Fig. 2). In older cats, the sutural gaps were smaller and contained a denser connective tissue with collagen fibres being orientated oblique to perpendicular to the suture line, with less osteoblasts and fibroblasts (Supplementary Fig. 2). There was no obvious difference in the micromorphology of the open sutures between the groups. In all examined specimens of peke-face kittens, a sutural gap between the parietal and frontal bones, i.e. the coronal suture was not present (Supplementary Fig. 1C). The morphology of the other sutures corresponded to that of DSHs and doll-face Persians.

Between the occipital and sphenoid bones, columns of proliferating and hypertrophic chondrocytes were seen, structured in a bidirectional manner from a central zone of resting cartilage. From the middle resting zone, layers of proliferative, columnar and hypertrophic chondrocytes emerged that are transformed to spongiosa at both ends. The phenotype of the spheno-ethmoidal synchondrosis differed from the others by its unipolar growth; a second ossification line within the cartilaginous nasal septum was not found. The single chondrocyte resting zone was located at the transition to the nasal septum. From here, chondrocytes undergo gradual transformation towards the presphenoid bone (Fig. 3 supplement). The width of the synchondroses gradually decreased from immature to older cats.

## Discussion

An extreme brachycephalic head morphology has become the most important breed‐defining trait in the modern Persian cat^1^, which is not found in the ‘old-fashioned’ type (‘doll-face Persian’) as it was bred for centuries^1^. In the late nineteenth century, the skull of the Persian was more and more modified through selective breeding of just a few mutated Persian cats with an unprecedented extreme short skull, displaying midface hypoplasia, frontal bossing, retrognathia superior and shallow orbits resulting in exophthalmos. This ‘peke-face Persian’ widely displaced the traditional ‘doll-face Persian’. A premature fusion of the coronal suture in Persian cats was suggested as the underlying cause for the extreme brachycephalic head morphology^10^. In the present study, we tested this hypothesis and evaluated the status of the calvarial sutures and synchondroses in a representative cohort of Persian and DSH skulls using MCT and histological examinations. The results of our investigation revealed that neurocranial sutures remain unfused in the majority of DSHs and doll-face Persians until old age. Histological examination showed that most suture sites represent a syndesmosis, in which the space between the cranial bones remains filled with connective tissue and few scattered fibroblasts. Ossification occurs individually in the last years of life with no clear influence of time. Only the sagittal sutures showed an age dependent ossification, with older DSHs having a higher probability of having a closed suture.

In humans, the calvarial sutures begin to fuse between the ages of 25–30 years. The sagittal suture closes completely by age 61–65, while the coronal suture is closed at the age of 56–60. The lambdoid suture fuses by age 66–70^11,12^. In other mammals, cranial sutures do not necessarily fuse and postnatal suture development is extremely variable. A syndesmosis between the skull bones is preserved in a lot of species that allows for a certain flexibility and energy absorption in the skull bones, which can reduce the risk of skull fractures in the adult animal^13–21^. Histological investigations of the calvarial sutures in domestic dogs also revealed that cranial sutures remain unfused representing syndesmoses until old age (> 12 years)^22^, which confirms the findings of the present study in cats.

While the metopic and lambdoid sutures were found to remain unfused until old age in the majority of peke-face Persians, the coronal sutures were ossified in all specimens, even in neonates. The shift in timing of a developmental event might occur as a physiological process in the evolution of skull morphology, which is referred to as heterochrony^23^. A longer or shorter growth period in cranial, and especially facial sutures, allowed for many evolutionary transformations and adaptions of skull morphology to species-specific feeding or breathing strategies^23^. The endpoint of coronal suture development in DSHs and the doll-face Persian, however, is not determined upon fusion but on transformation into a syndesmosis. The true ossification of the coronal suture in immature peke-face Persians is therefore very unlikely to be a heterochronic process. During the first year of life, the skull vault expands until the brain reaches its mature volume. Brain growth continues until at least six months post-partum in cats^24,25^ and the skull must adapt to brain expansion during this time. A cessation of growth in the coronal sutures has a severe impact on cranial capacity and results in severe alterations of the brain in peke-face Persians^10^. Furthermore, we found Wormian bones (sutural bones; supernumerary bones) in the metopic, sagittal and lambdoid sutures of peke-face Persians. Wormian bones represent independent centres of ossification that develop in association with unphysiological stress and load on the calvarial sutures, such as those that occur in the course of pathologic skull deformation^26^. Wormian bones are mainly observed in humans with craniosynostosis and in laboratory rabbits with the same growth defects^21,27–29^. Together with the observed diastasis of the other sutural gaps and the relatively large occipital bone, it can be assumed that compensatory overgrowth occurs at the non-fused sutures to compensate for the absent skull vault growth in the coronal sutures.

While the cranial sutures remain unossified, skull base synchondroses regularly undergo ossification and fusion in all eutherian and metatherian species^30^. The time point of ossification has interspecific and interindividual variation^16,31^. Thyroid^32^ and sexual hormone status^33^, as well as duration of general skeletal growth^34^ have a major influence on the individual postnatal ossification of the synchondroses. Skull base synchondroses of both peke-face and doll-face Persians ossified between 12 and 14 months, while the synchondroses of the DSHs ossified much later (18–29 months). It is possible that the skull morphology of the original doll-face Persian is based on a reduced longitudinal growth of the skull base, caused by an earlier fusion of the skull base synchondroses as it is known from brachycephalic dogs^10^. As the peke-face Persian arose out of the doll-face Persian, both phenotypes might share this common skull base growth defect and the ossification of the coronal suture occurred as an additional change in skull growth that further modified the skull of the cats to its present appearance.

The premature fusion of one or more cranial sutures is defined as craniosynostosis, which encompasses an array of conditions in humans that all affect the growth of the cranial vault. It can affect a single suture or occur in variable patterns of suture closure combination^35^. Furthermore, it may occur as part of a syndrome affecting other organ systems (syndromic craniosynostosis, such as Crouzon, Apert, Saethre-Chotzen and Pfeiffer syndromes) or in isolation (non-syndromic craniosynostosis)^36^. Most children with coronal craniosynostosis share a common phenotype with a dorsally round and dome-shaped skull, frontal bossing, increased interorbital distance (hypertelorism), ocular proptosis as well as a hypoplastic maxilla and prognathic mandible, resembling the phenotype in the peke-face Persian^10,37,38^. Defects in other organ systems (syndactyly, fusion of the cervical vertebrae, cardiovascular defect, cleft palate, brachydactyly, acanthosis nigricans, hyperpigmentation of the skin, tarsal fusion, sensorineural hearing loss, etc.)^39^ are not reported in the modern Persian cat, which is why craniosynostosis is most likely non-syndromic in this species. Closed sutures were found in male and female cats, which rules out any X-linked disorders (i.e. craniofrontonasal syndrome)^40^. Coronal craniosynostosis is found in humans with mutation genes encoding for MSX2 (Boston type craniosynostosis), TWIST and TCF12 (Saethre-Chotzen syndrome), as well as FGFR3 (Muenke craniosynostosis)^41^, which could be interesting candidate genes for further investigations of a possible underlying genetic defect in peke-face Persians.

Despite increasing information about health issues that are related with the brachycephalic phenotype, a reconsideration of breed standards in Persians and other brachycephalic cats has not yet occurred^42^. Based on the results of this study, breeders and cat fanciers must understand that the desired morphology of the head in the modern Persian cat is not only associated with health issues, but that the desired skull phenotype itself is based on a disease process, i.e. a craniosynostosis that causes the above-mentioned devastating diseases in children. The breed standard for Persian cats does not describe natural morphologic traits but pathologic clinical signs that are the consequence of a pathologic skull development^5,10^. According to German Animal Protection law, it is prohibited to breed animals with defective organ systems. The above described coronal craniosynostosis fulfills this condition to the greatest possible extent. As a logical consequence the selection for extreme forms of brachycephaly in Persian cats is a clear violation of German Animal Protection Law.

## Conclusion

In conclusion, a coronal craniosynostosis causes the particular skull morphology of the peke-face Persian. The naturally occurring craniosynostosis in cats may represent a unique opportunity to investigate the pathophysiology and therapeutic intervention in a large animal translational model beyond genetically modified rodent models.

## Supplementary Information

Supplementary Figures.

## Data Availability

All data generated or analysed during this study are included in this published article.

## Abbreviations

CS: Complete separation
DSH: Domestic Shorthair cat
MCT: Micro-computed tomography
μm: Micrometre
μA: Micro ampere
mm: Millimetre
S1: Sagittal suture
S2: Metopic suture
S3: Left coronal suture
S4: Right coronal suture
S5: Left lambdoid suture
S6: Right lambdoid suture
S7: Intersphenoidal synchondrosis
S8: Spheno-occipital synchondrosis
S9: Spheno-ethmoid synchondrosis
