# Supplementary material for: Closure times of neurocranial sutures and synchondroses in Persian compared to Domestic Shorthair cats
Source: Sci Rep. 2022 Jan 12;12:573. doi: 10.1038/s41598-022-04783-1 (PMC8755779; doi:10.1038/s41598-022-04783-1)
Supplement: Supplementary file 1 — Supplementary Figures. [file 41598_2022_4783_MOESM1_ESM.pdf]

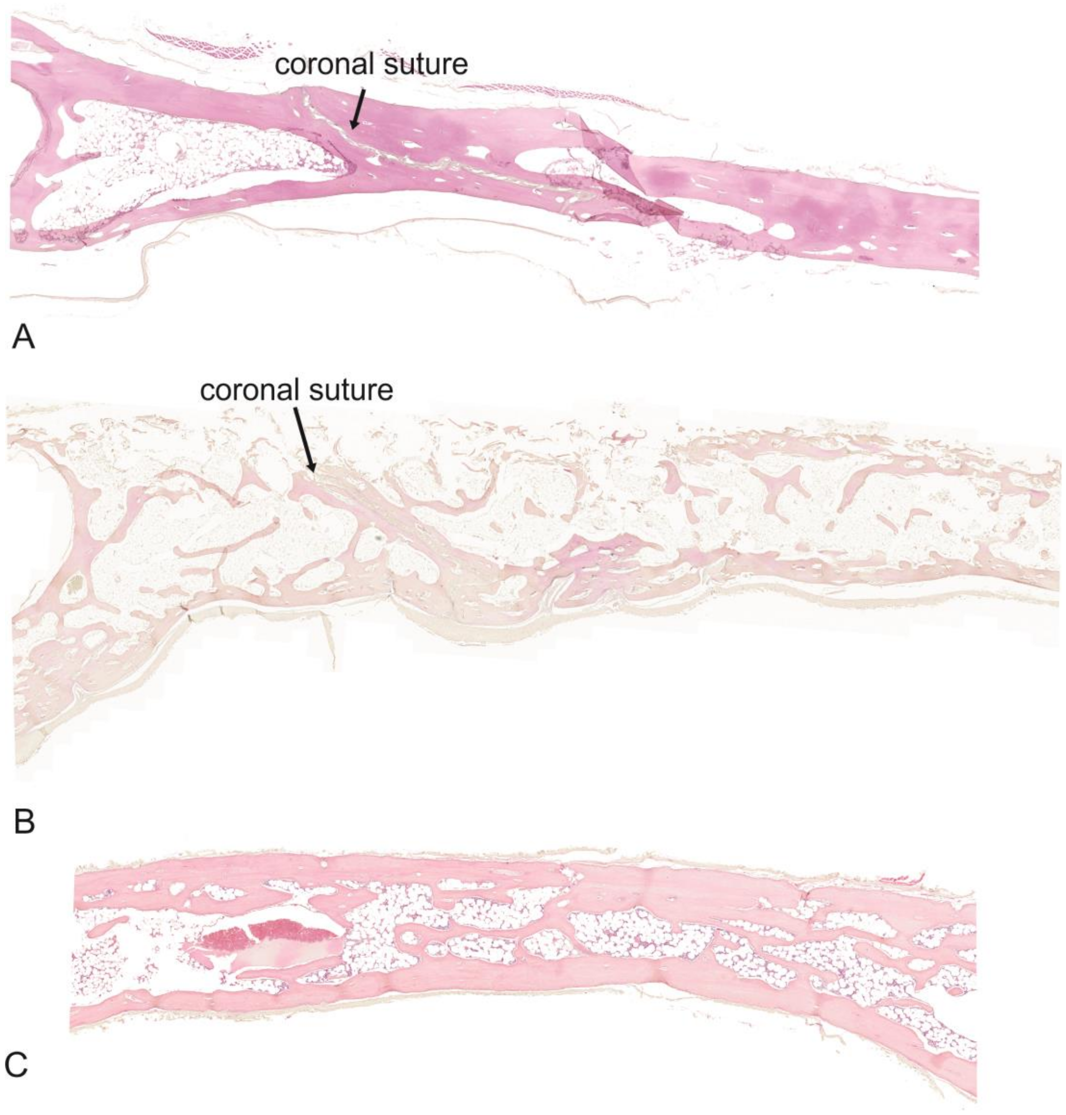

Figure 1 supplement: Sagittal section of the frontal and parietal bones of the skull of a two-week-old DSH kitten(A), a four-year-old DSH (B) and a three-day-old peke-face Persian (C): haematoxylin and eosin stain. The coronal suture can be clearly seen as an oblique gap between the frontal and parietal bones, filled with fibrocytes and collagen fibres. A coronal suture is not present in the peke-face Persian.

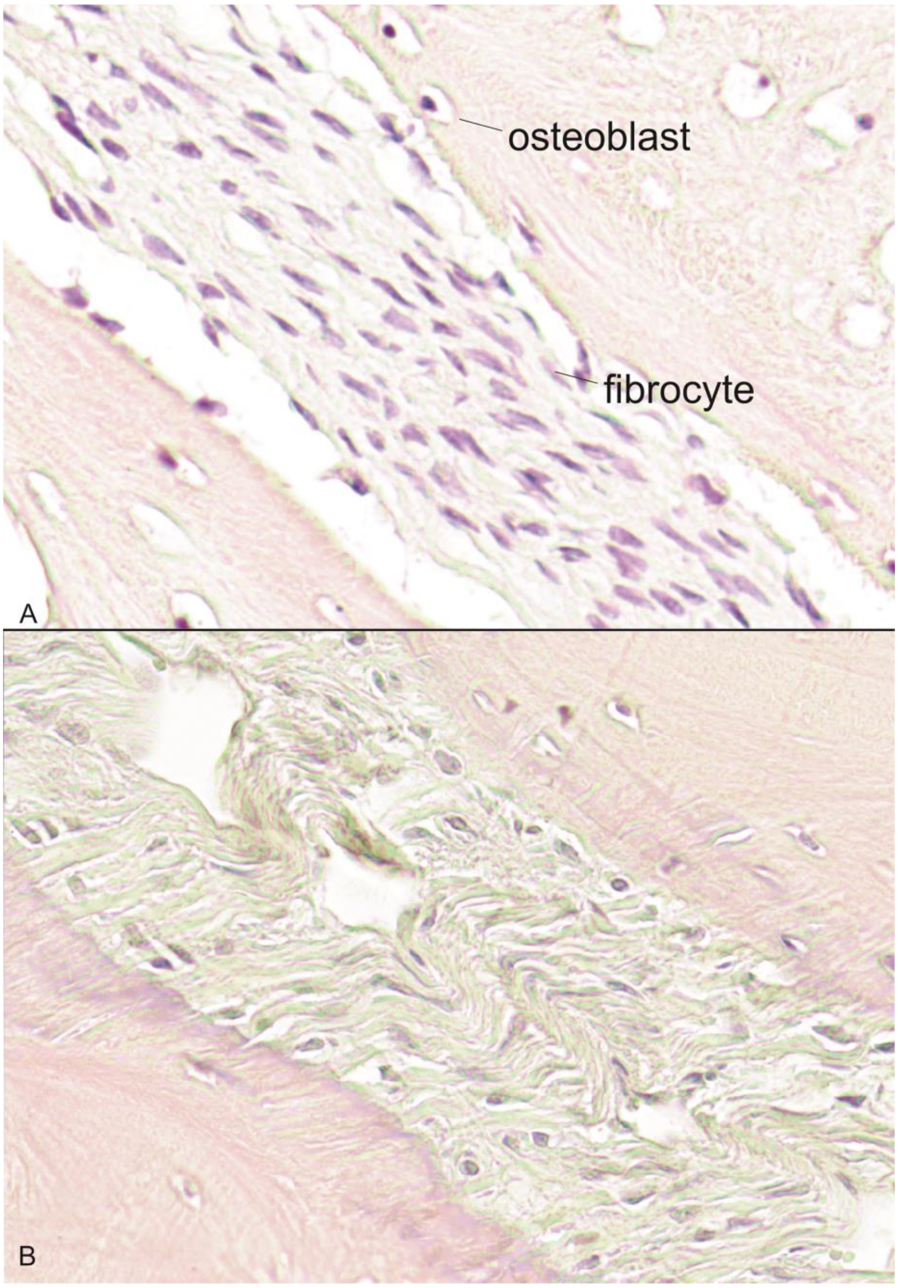

Figure 2 supplement: High magnification image of the coronal suture of a two-week-old DSH kitten (A), a four-year-old DSH (B): haematoxylin and eosin stain. In the immature suture, fibroblasts in a collagen-rich matrix dominate the gap between bone plates. Collagen fibrils have no preferential orientation. Scattered osteoblasts embedded within the mineralising matrix line the suture gap (A). The number of fibrocytes is reduced in the syndesmosis (B). The two borders of the suture are interconnected by a dense network of collagen fibres arranged perpendicularly to the osseous borders. They are continuous with Sharpey's fibres within the borders of the coronal suture.

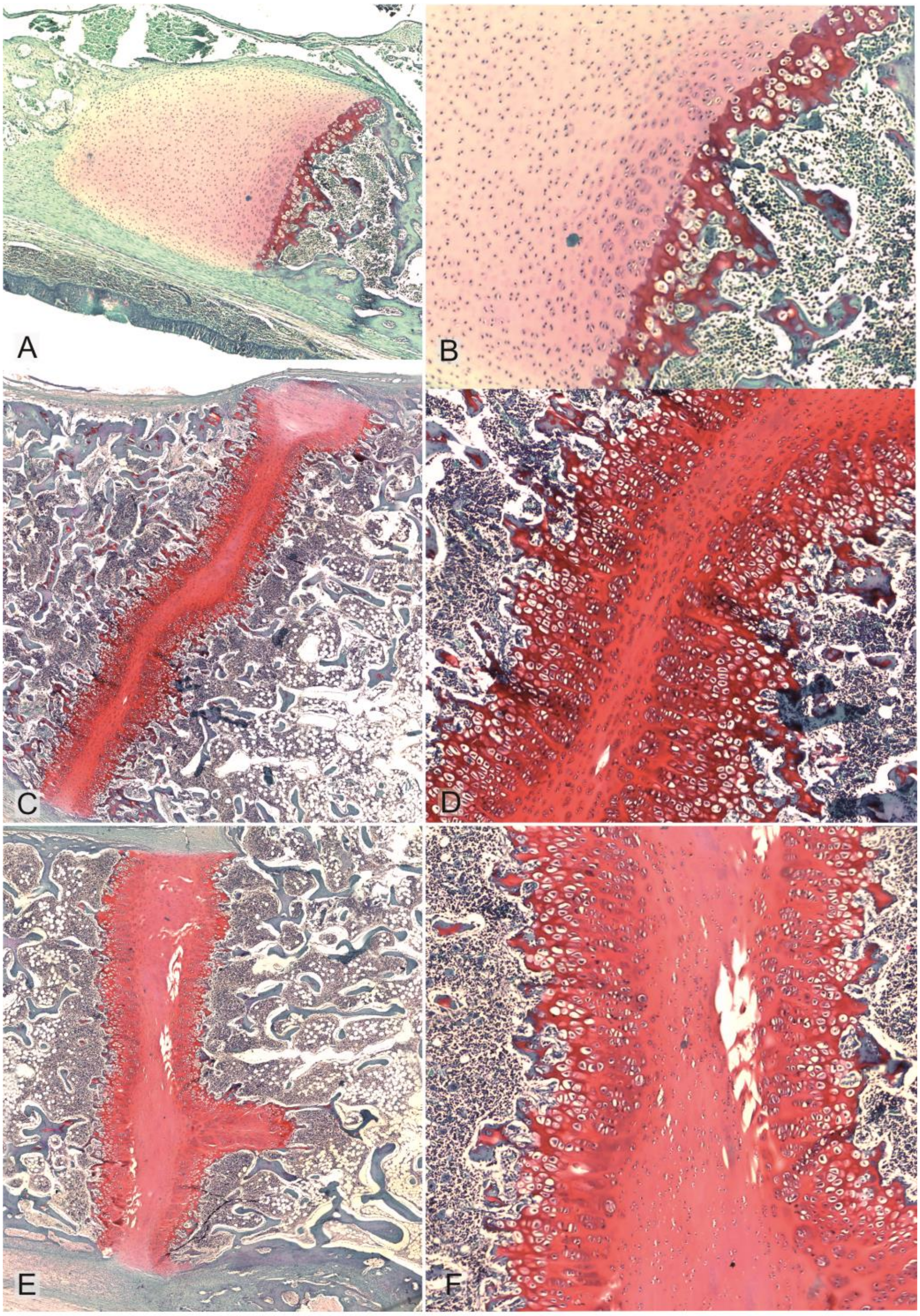

Figure 3 supplement: Sagittal sections through the spheno-ethmoid (S9), intersphenoidal and spheno-occipital synchondroses of a four-month-old DSH: Masson-Goldner trichrome stain. Columns of proliferating and hypertrophic chondrocytes are seen, structured in a bidirectional manner from a central zone of resting cartilage (C, D, E, F). From the middle resting zone, layers of proliferative, columnar and hypertrophic chondrocytes emerge that are transformed to bone spongiosa at both ends. The phenotype of the spheno-ethmoid synchondrosis (A, B) differed from the others by its unipolar growth; a second ossification line within the cartilaginous nasal septum is not present. The single chondrocyte resting zone is located at the transition to the nasal septum. From here, chondrocytes undergo gradual transformation towards the presphenoid bone.
